# Scaling up antiretroviral therapy in Uganda: using supply chain management to appraise health systems strengthening

**DOI:** 10.1186/1744-8603-7-25

**Published:** 2011-08-01

**Authors:** Ricarda Windisch, Peter Waiswa, Florian Neuhann, Florian Scheibe, Don de Savigny

**Affiliations:** 1 Swiss Tropical and Public Health Institute, Basel (P.O. Box 4002), Switzerland; 2 University of Basel, Basel (P.O. Box 4003), Switzerland; 3 College of Health Sciences. School of Public Health, Makerere University, Kampala (P.O. Box 72515), Uganda; 4 Demographic and Health Surveillance Site, Makerere Iganga-Mayuge (DHSS), Kampala (P.O.Box 7072), Uganda; 5 Institute of Public Health, University of Heidelberg, Heidelberg (69120), Germany

## Abstract

**Background:**

Strengthened national health systems are necessary for effective and sustained expansion of antiretroviral therapy (ART). ART and its supply chain management in Uganda are largely based on parallel and externally supported efforts. The question arises whether systems are being strengthened to sustain access to ART. This study applies systems thinking to assess supply chain management, the role of external support and whether investments create the needed synergies to strengthen health systems.

**Methods:**

This study uses the WHO health systems framework and examines the issues of governance, financing, information, human resources and service delivery in relation to supply chain management of medicines and the technologies. It looks at links and causal chains between supply chain management for ART and the national supply system for essential drugs. It combines data from the literature and key informant interviews with observations at health service delivery level in a study district.

**Results:**

Current drug supply chain management in Uganda is characterized by parallel processes and information systems that result in poor quality and inefficiencies. Less than expected health system performance, stock outs and other shortages affect ART and primary care in general. Poor performance of supply chain management is amplified by weak conditions at all levels of the health system, including the areas of financing, governance, human resources and information. Governance issues include the lack to follow up initial policy intentions and a focus on narrow, short-term approaches.

**Conclusion:**

The opportunity and need to use ART investments for an essential supply chain management and strengthened health system has not been exploited. By applying a systems perspective this work indicates the seriousness of missing system prerequisites. The findings suggest that root causes and capacities across the system have to be addressed synergistically to enable systems that can match and accommodate investments in disease-specific interventions. The multiplicity and complexity of existing challenges require a long-term and systems perspective essentially in contrast to the current short term and program-specific nature of external assistance.

## Background

**Figure 1:**
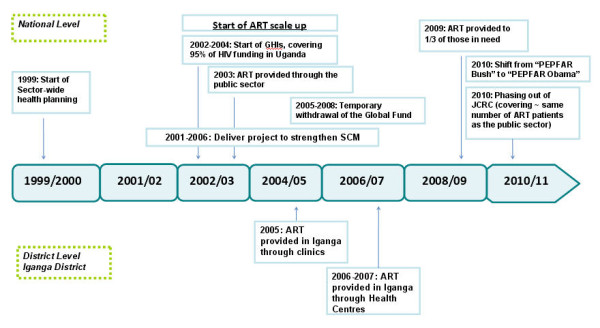
**Major events during antiretroviral scale-up in Uganda**.

The scaling up of antiretroviral therapy (ART) in Uganda gathered momentum with three major global health initiatives (GHIs): the Multi-Country HIV/AIDS Program (MAP) in 2002; the United States President's Emergency Plan for AIDS Relief (PEPFAR) and the Global Fund to Fight HIV/AIDS, Tuberculosis and Malaria (GFATM) in 2004. Free antiretroviral drugs (ARVs) have been provided in the public governmental since 2003, when the first national ART strategy and treatment guidelines were developed [1-3]. Figure 1 illustrates the main events in Uganda as they concern the expansion of ART.

By the end of 2009, 200,400 people were receiving antiretroviral therapy and coverage of those in need based on the new 2010 World Health Organisation (WHO) thresholds had reached 39% [4]. In terms of numbers the country has consequently come relatively close to its targets of 240,000 and 342,200 people on treatment by 2012 and 2020. However 95% of that national response to ART is currently covered by donor funds [5]. Uganda, as it is estimated for other low-income countries, will continue to depend largely on external support for its disease-specific programs [6-8].

Given that ART and its supply chain management in Uganda are today mainly based on parallel and externally supported efforts, the question arises for how to sustain these once government is required to take over. Uganda is starting to face that reality in the transition of PEPFAR from the Bush to the Obama administration and plans [9]. Sustained access to ART will essentially depend on the strength of health systems. Looking at some core indicators, the country's skilled birth-attendance rate is 42%, its measles immunization rate for 1-year-old children is 68% and malaria-treatment access within 24 hours of fever for children under 5 is 35.7% [10,11]. As is the case in other low-income countries, supply chain management is an especially weak part of the national health system. The essential drug program lacks more than 50% of the funding it would need for the constant supply of the minimum care package [12]. Only 27% of hospitals and about 40% of other health facilities report receiving the requested quantities of essential drugs ordered through the National Medical Store (NMS) [13]. Likewise and despite its relatively high external support antiretroviral drug supply experiences both over and undersupply [14]. Weak health systems appear to constrain absorption of external funding. Only 26% of a Global Fund grant in Uganda had been spent after twenty months [15,16].

Extensive literature reviews have summarized findings about the effects of GHIs on health systems [17-19]. Research has, however, focused on single effects and paid little attention to the interactions among health system building blocks and interventions or the role of contextual and governance issues [20-24]. Systems thinking is a key approach to illuminate what works, in what way and for whom, in a given context. It also serves to explore the range of effects and potential synergies, causal chains and linkages between complex interventions such as ART and health systems [20].

To address these issues, we apply systems thinking to the case of supply chain management for ART in Uganda. We use the WHO health systems framework and examine dimensions of governance, financing, information, human resources and service delivery in relation to supply chain management for ARVs and essential drugs. This paper takes the viewpoint of a close examination of consequences at district levels, and traces their causes within the governance and other building blocks of health systems.

## Methods

This work uses findings from document and literature review, health facility surveys, and key-informant interviews at district and national levels. A literature review was conducted covering both peer-reviewed and grey literature, including the media. Sources included PubMed, Web of Science, Eldis, Google and Google Scholar. Grey literature such as audit reports, evaluations and tracking studies were a main source of information. National level assessments were based on principles of Grounded Theory implying that the process of data collection and emerging findings continuously shape research approaches [25-27]. A first question guide focused on information gaps which resulted from the review. National partners performed key-informant interviews, based on a few guiding questions which allowed respondents to flexibly raise new issues and hypotheses. To ensure consistency of interpretation, interviews were conducted by the investigators themselves. Responses were validated in subsequent interviews with other stakeholders. We triangulated the different sources for validation by following up findings from the literature review and within interviews and relating findings at district and facility level with views from national stakeholders.

Observations at health service delivery level took place in Iganga District in the Eastern Region of Uganda. The study site Iganga was chosen as it is also the study site of a larger research project studying the effects of antiretroviral treatment on maternal and child health. Iganga is one of 95 districts in Uganda and it covers a mainly rural area with a population of around 650,000 out of the national population of 32.4 million [28]. Four health centres (HCs) at level IV and III and one district hospital provide ART services. HC-IVs are structurally small clinics with 1-2 clinicians, an obstetric theatre and laboratories. HC-IIIs also provide some laboratory services. The district hospital started to provide ART in 2005 followed by gradual provision through HCs in 2006 and 2007. By September 2009 a total of 1,171 people in the district had been started on antiretroviral drugs (ARVs). To evaluate the performance of ART at the service-delivery level in Iganga District, two onsite surveys were conducted at all ART-providing HCs in June 2008 and September 2009. They included a complete document review of registers, logbooks, drug stocks, patient files and observed practices, and staff and patient interviews in 72 health facilities. Semi-structured interviews were conducted with 17 health staff and 273 patients. The detailed results will be published in a separate paper currently in process.

## Results

### Supply management systems

Essential drug supply in Uganda uses a mixed "push" and "pull" system. Upper-level health facilities order drugs based on estimated need forecasts and a resource envelope. Lower-level health facilities receive a fixed set of drugs. The essential drug list includes 96 drugs for districts to order from the National Medical Store (NMS), which processes almost 1,000 individual orders per month. When ART started, supply chain management systems for essential drugs had just started to be built to reach national coverage through a pull system. Drug delivery to districts can take about double the time foreseen [29]. One of the bottlenecks was that the NMS only delivered to district headquarters. Since 2009 the NMS also delivers to HC IV and III level [30]. Faith-based and non-governmental organizations (FBOs and NGOs) which account for 20-30% of the health facilities in Uganda are served through a cash-and-carry system of the Joint Medical Store (JMS). The NMS procures and manages an increasing number of ARV drugs and supplies, 46 different ARV drugs and drug combinations were registered in 2003 [31]. ARV procurement and supply runs through standard NMS processes such as the bimonthly essential drug delivery as well as on parallel processes specifically set up for ARVs. The latter generally works better due to more funding and smaller volumes [32].

**Figure 2:**
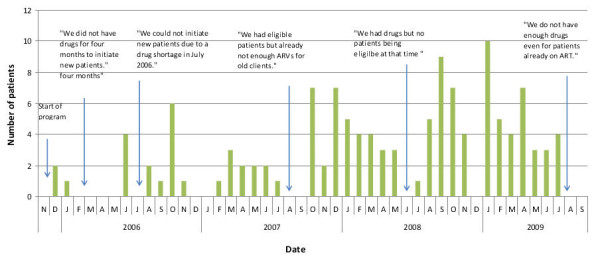
**Fluctuations of number of new patients on ART and their causes**.

At Iganga District ARV shortages affected all ART-providing facilities with considerable fluctuations regarding capacities to take up new patients as illustrated in Figure 2. ARVs were available at 83%, diagnostic kits at 70% and paediatric ARVs at less than half of the health facilities surveyed. Stock-outs also occurred for antibiotics, including amoxicillin and cotrimoxazole dispensed as prophylaxis for opportunistic infections in HIV-positive patients. Effects included problems in patient follow-up and in the provision of ART. Patients were advised to buy missing drugs in private pharmacies. Switches to more complex and different drug regimens were frequent to avoid treatment interruptions. Strategies to cope with stock-outs included lending and borrowing among facilities, duo-therapy, late initiation of ART for new patients and treatment interruption. ARV regimens from ten different manufacturers were found. Health workers reported insufficient knowledge regarding safe drug substitution and a general lack of guidance to deal with shortages of ARVs. They faced difficulties in forecasting needs given the lack of data. District medical officers (DMO) were bypassed as facilities communicated directly with the NMS. Lack of feedback from the NMS on placed orders further reduced their capacity to address potential bottlenecks.

National level surveys substantiate that provision of ARVs suffers from both over and undersupply. According to findings from 2007 only a quarter of facilities receive ARVs on a monthly basis, which is the required frequency for consumption reporting [33]. At the same time USD 0.5 million of ARVs are reported to have expired in 2005 [34]. In 2008 the estimated expired value was in the range of USD 1.3 - 2 million [35]. 58% of government facilities reported holding expired ARVs, compared to 29% of NGO facilities [33]. Test kits, prophylactic treatment and paediatric ARVs are especially affected by short supply. According to a health facility survey in 2005 fewer than 25% of facilities were maintaining adequate stock levels on nevirapine, HIV test kits, and antibiotics to treat opportunistic infections (OI) and sexually transmitted infections (STIs) [34]. Health facilities on average reported 1 month of stock-outs of testing kits per year in 2005 [14]. Undersupply of test kits was mainly caused by unexpected supply disruptions from two donors and resulted in rationing with a focus on preventing mother-to-child transmission (PMTCT) clients instead of the general population. Findings from 2008 suggest that some facilities faced shortages over several months. Only about 15% of patients in need could be tested as a consequence [36]. A 2004 national laboratory assessment indicated that due to a lack of reagents, half of the regional hospitals could not perform confirmatory diagnostics for OI and 20-30% of district hospitals could not perform basic STI and OI diagnostic tests [37].

For essential drugs, despite a four-fold increase in the value of drugs distributed, less than half the money needed for the basic minimum care package is available. This means that most drugs will always be stocked out because of insufficient funds as opposed to supply chain problems [12]. Only 27% of hospitals and about 40% of other facilities reported receiving the quantities of essential drugs they ordered through the NMS [13]. Improvements in some areas exist such as an increase of available drugs for STIs from 8% in 2002 to 24% in 2006 [34].

**Figure 3:**
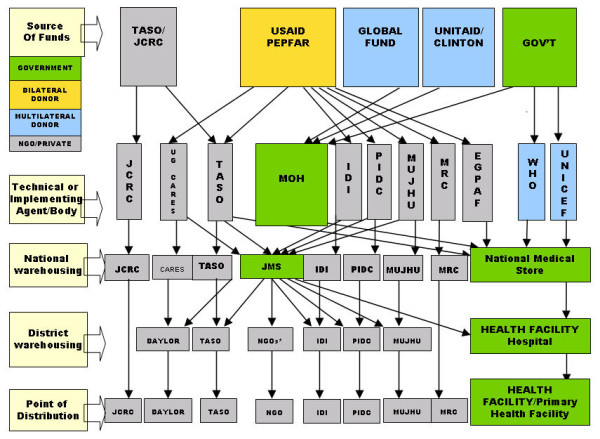
**Antiretroviral supply system in Uganda, 2010**. IDI Infectious Diseases Institute. JCRC Joint Clinical Research Centre. JMS Joint Medical Store. MRC Medical Research Council. MUJHU Makerere & John Hopkins University Research Collaboration. PIDC Paediatric Infectious Diseases Clinic. TASO The AIDS Support Organisation. * Some NGOs also deliver to government health facilities.

Figure 3 shows the number of largely externally supported systems to supply ARVs. It illustrates procurement, storage and distribution systems for ARVs in the country with nine different lines of procurement and supply for these drugs alone. PEPFAR, for example, requires the US Food and Drug Administration approval of ARVs instead of the WHO prequalification commonly used by other donors and countries [38]. It also specifies selected ARV manufacturers and therefore constrains use of local ARV production which Uganda started in 2008 [29]. Most GHIs use the national governmental system for drug storage and distribution. NGOs funded by PEPFAR, however, follow their own storage and distribution systems. Overall, external support focuses on narrow, short-term and parallel approaches. PEPFAR initiatives largely target the Non-governmental and Faith-Based Organization sector with only some indirect support to the MoH, mainly providing training and laboratory equipment [36]. All GHIs support warehouse capacity and short-term training. The Global Fund has to some extent taken a more systems-based approach by increasing human resource capacity through the funding of procurement officers [32].

An initial policy intention existed to assimilate ARVs with the essential drug supply system. Procurement was meant to be aligned; ARVs were meant to be included in the essential drug list; and a logistics management information system (LMIS) for ARVs was intended to be put in place [31]. However, as existing supply systems were considered too weak to support the national ART program, separate systems were set up with the objective to integrate them later at an unspecified date [12]. Parallel supply chains have gained additional leeway due to free choice of private facilities to choose logistic providers and similar options for public facilities sectors if the NMS does not deliver. These parallel options were justified on the grounds of a need to initially strengthen the NMS [39]. A main initiative to support NMS' capacities was the DELIVER project from 2001-2006. DELIVER however at the end of the day also supported parallel supply chain management systems of NGOs such as the Joint Clinical Research Center (JCRC), a PEPFAR-funded NGO which covered almost half of the patients on ARVs in Uganda until it started to phase out in 2009. Another policy intention to address inefficient ARV supply was issued in 2008 when the government expressed a target of reducing yearly expiration of unused drugs to a maximum of USD 1000 annually by, for example, denying superfluous or non-aligned external funding as well as improving the information system for drug supplies [35]. No progress on these initiatives was documented at the time of this study

### Governance

External actors very much shape current governance of ARV supply chains. In Iganga District 15 NGOs were found to work in the area of HIV; two of them being directly involved in ART. Perceptions at district level are that there is generally little cooperation between NGOs themselves and the health district. Usually no joint planning efforts take place. District health managers often lack information on projects and links of NGOs. At national level, integrative efforts were already lacking prior to ART as sector-wide planning in the health sector only started in 1999. Surveys of the Country Coordination Mechanism (CCM) of the Global Fund, for example, present a relatively large and inefficient committee, whose role partly covers that of the Ugandan AIDS Commission (UAC). PEPFAR has a policy to mainly support NGOs, the majority of which are based in the capital Kampala and relatively distant to district levels. In some measure they were found to be part of the problems related to poor accountability which lead to the temporary suspension of Global Fund grants in 2005 [40].

Poor accountability and mismanagement is another governance issue for drug supply. At district level funding for essential drugs is not always used according to guidelines. Districts often do not include the purchase of lab supplies in their budgets as required [31]. HCs are often not aware of how much funding for drugs is credited to their accounts. In one district almost half of the budget for essential drug purchase was not utilized and two thirds of unused funds could not be accounted for in the fiscal year (FY) 2004/05 and 2005/06. In the FY 2000/01 USD 1.75 million remained unspent in district health accounts [34]. The average leakage rate for drugs across ten public health facilities in Uganda was estimated at 73%, with lowest availability of high demand drugs, such as those to treat malaria [41]. Some physicians are alleged to reroute essential drugs to private clinics and pharmacies and then send public patients to these outlets to purchase their medication. They may also under-procure drugs to cause a shortage which is then covered by the private market. Mechanisms to regulate are made dysfunctional as the district planning teams responsible for monitoring are sometimes involved in these diversions for private health care [30].

Parallel to ART scale up an increasing number of national frauds or mismanagements occurred. USD 190,300 earmarked for drugs was for example used for travel abroad for government officials in 2006 [42]. In another case three former health ministers and other ministry staff were charged with alleged misappropriation between 2006 and 2007 [43]. The Global Fund suspension in 2005 resulted in some initiatives to correct for non-compliance but disbursements did not resume until 2008. That year encountered another case of poor accountability resulting in a Global Fund disbursement gap of USD 12 million [44]. The government mobilized USD 30 million to fill the most severe shortfalls, but could not completely avoid service delivery effects such as stock-outs of antimalarials [45].

### Financing

Bypassing, inadequate funding and dependency on external donors were identified as main constraints to better performance of the NMS [35]. Reimbursement modalities were not defined when the NMS received the logistics mandate for ARVs in 2003. The NMS usually requires 6-10% ordered to cover storage, handling and distribution. While programs usually pay 10%, MAP, for example, only paid 6.5% arguing that the lower percentage is justified given the high value of ARVs. Another issue is that being a public agency, the NMS deals with relatively long lead times in procurement, which is one of the reasons why donors have opted for other procurement channels [31].

External funding will continue to affect access to ART. Funding for ART has increased considerably, but remains unstable and unpredictable. Global Fund moneys for HIV increased by 45% between 2004 and 2005 and then dropped by 18% following its temporary suspension in 2005 [46]. PEPFAR's share of HIV funding in Uganda increased from 26% in 2003 to 85% in 2006 [16]. Predictions envisage decreasing funding due to expressions of the US government to scale PEPFAR down and hand over responsibilities to national governments [9].

### Human Resources

National level data confirms a severe lack of human resources in the area of supply chain management. While the public sector in Uganda has about 350 qualified pharmacists, it is estimated that at least 14,000 are needed [29]. One of the reasons is a high turnover of pharmacists, who go abroad or work in the private sector. A perception at national level is, for example, that PEPFAR recipients have attracted the best health workers from the government systems, especially doctors and higher cadre nurses [40]. Salaries are much higher within externally funded projects. Salaries of nurses and doctors working for PEPFAR-funded programmes for example are more than twice as high as those in the public sector [40].

### Information Systems

Figure 3 shows the number of supply chain management programs and their information systems. Our Iganga District assessment revealed a range of parallel information processes due to external initiatives. JCRC for example, despite its policy to use Ministry of Health (MoH) forms, was using separate forms. Obstacles resulted when patients transferred to the public system in 2009. Different coding systems and discontinued files also contributed to misinterpretation of drug consumption rates needed to inform the drug orders. Instructions on new patient files and documentation remained poorly communicated to succeeding programs. The Iganga surveys also showed poor local compliance with information requirements. Three out of five sites handled the filing of patient cards poorly. Files were not kept in a way that allows easy retrieval and had to be sorted before assessment. The district as a consequence misses the data needed for its supply forecasts, including numbers lost to follow-up.

National level surveys corroborate these findings. One highlights a general lack of stationery, outdated forms, superfluous and duplicated reporting requirements, incoherence in indicators as well as inconsistency between systems that rely partly on computers, partly on manual filing. Effects are weak processes, incomplete record, file-keeping and reporting, the loss of data as it is being aggregated from district to national level, and non-use of composed information [32]. Another survey specifies weak inventory management of laboratory commodities, half of the facilities did not use any report forms and only about a quarter used stock cards [37]. Other research shows distorting effects such as oversupply in cases where MoH and PEPFAR-funded NGO projects deliver drugs to the same facilities and patients [32].

The national policy in 2003 was to merge the HIS for ART with the national LMIS: Logistic Management Information System (LMIS) and the overall national Health information system (HIS) [39]. A first barrier was that national ART programs were at the outset based on parallel LMISs. In 2004 three major systems existed: One for the MoH free provision of ARVs and two for JCRC that distinguished between free and sold ARVs. The LMIS and HIS for essential medicines are yet not integrated. One of the reasons is that clinical care and drug logistics are managed by different committees that would need to coordinate efforts [31]. This lack of well developed and integrated national HIS has triggered further development of parallel HIS for ARVs [47]. The disadvantages of that trend were recognized, but perceived as necessary to reduce the risks associated with the high costs of ARVs. So far only a few isolated efforts to centralize information on logistics have materialized, such as incorporating ARV logistic forms into the national HIS [12]. The need for an LMIS system covering all essential drugs continues to be on the agenda but has not received adequate funding and political support [31].

### Service delivery

Stock outs at the point of service delivery are critical indicators of poor quality services from the client perspective. Not all stock outs are supply chain management related per se. Previous sections covered these manifestations of service delivery as they directly relate to supply chain management. Many other elements of service delivery may result in lack of drugs and supplies which are not directly related to supply chain management, including for example adequacy of infrastructure and human resources in general. Important shortages exist in areas such as laboratory equipment and reagents. A 2006 health facility survey found most health facilities lack essential laboratory equipment [34]. According to another survey only 17% of the HC counselling rooms for HIV complied with national guidelines. While all health centres providing PMTCT and voluntary counselling and testing (VCT) have laboratories for testing, technicians were not always available [14]. Condoms were the least available contraceptive assessed during a health facility survey in 2006, resulting in a stagnating contraceptive coverage is stagnating at 23% [34]. Shortages were fuelled by a MoH policy to withdraw condoms from facility level in order to introduce quality assurance for all incoming condoms which caused supply disruptions for 1.5 years [48]. Between 2002 and 2006 family planning methods have only increased from 24% to 35% [34].

## Discussion

Our assessment of the supply chain management at Iganga District indicates important bottlenecks and system failures. We examine these through a systems thinking approach linking dynamics and causes across different sub-systems at district, national and international level. Poor performance of supply chain management is being reinforced by poor conditions at all levels of the health system, including the areas of financing, governance, human resources and information. Table 1 summarizes the range of systems features as they relate to different building blocks. Systems weaknesses are the main reasons why - despite initial policy intentions to opt for integrated approaches - parallel systems are being built that increase complexity and trigger inefficiencies. Poor performance results in less than satisfactory delivery not only for ART but for health service delivery in general. Shortages are particularly apparent for drugs and supplies other than ARVs. In Iganga the supply of cotrimoxazole for example by did not match by far the needs generated by ART expansion. Essential drugs and supplies shortages also show how, at a time of complex endeavors to deliver ART, many other essential and more affordable and cost-effective health services still fall short of supply. Many higher burden problems remain neglected by GHIs such as childhood pneumonia and maternal mortality which appear to be particularly affected by relatively little attention and funding [49,50].

**Table 1:** System effects of ART expansion in Uganda

System Outcomes	Description of System Causes and Effects	Primary Sub-system affected
More people on ART	The country has rapidly expanded ART with a 50% coverage of those in need by the end of 2009. Effects include creation of demands that require the systems to sustain an appropriate level of care.	Service delivery, with knock-on effects on all other sub-systems

Supply shortages (essential drugs) and expiry (ARVs)	Little investments in strengthening supply systems for essential drugs, lack of qualified staff leading. Effects include poor health outcomes, inefficiencies, financial and credibility losses.	Technologies, with knock-on effects on all other sub-systems

New supply chain management systems and governance structures for ART	Interest for short-term targets easier achieved through parallel systems. New structures and interests difficult to readjust later on. Effects include poor outcomes, vicious circles between weak systems and vertical approaches.	Governance, Technologies, Information, as well as the other sub-systems

ART program related mismanagement	Partly due to lack of absorptive capacity for rapid and large funding. Effects include misappropriation, withdrawal of funding, inefficiencies.	Governance, with knock-on effects on all other sub-systems

Brain drain, lack of qualified and motivated staff	Focus on short-term trainings, lack of training, higher salaries and other incentives within disease-specific programs compared to the public sector	Human Resources, knock-on effects on all sub-systems

Lack of appropriate data	Parallel, partly inefficient as well as unfeasible programme specific information systems. Effects include failure to focus on one national information system that meets quality standards, inefficiencies, superfluous tasks at facility level.	Information, knock-on effects on all sub-systems

Findings from other countries substantiate the trends seen in this research. A study in six Sub-Saharan African countries shows that counterfeits and sub-standard drugs are becoming commonplace [51]. Surveys on health system effects of disease-specific programs unanimously report adverse effects in the area of governance with parallel bureaucracies, a general lack of aid coordination and integration to national systems [7,15,17,52-62]. Common themes related to supply chain management include donor driven priorities and systems, unwieldy procedures, uncoordinated practices, negotiations with different donors, excessive demands on time, different funding mechanisms and reporting expectations as well as delays in disbursements [63-65]. In Malawi procurement guidelines of the World Bank were used despite being perceived as cumbersome [66]. In Benin and other countries little attention has been paid in strengthening government procurement capacities [56].

**Figure 4:**
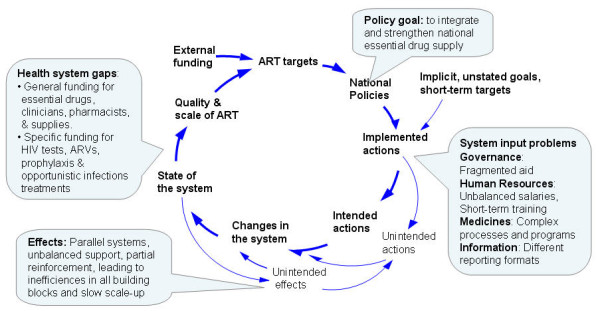
**System dynamics of supply chain management for ART**.

Governance of drug supply chains appears as a key driver of systems performance. This research highlights important gaps between stated intentions, policies and implementation. Figure 4 illustrates the dynamic relationships between external inputs, intended and unintended actions at different dimensions of the health system as conceptualized by systems thinking [23]. External actors follow their own agendas, set up parallel processes and follow short-term approaches. External initiatives focus on "easy" bottlenecks, such as clinical knowledge and warehouse capacity and avoid the more complex issues of systems strengthening [67]. As a MAP official put it: "We somehow strengthened the supply chain but it was temporary; no efforts continued after the project closed" [32]. Exceptions such as the DELIVER project exist but remain inhibited by system constraints. Government lacks administrative capacities, regulatory structures, information and incentives needed to monitor and ensure quality standards. These system constraints constitute common weaknesses in low-income countries [68,69]. Poor accountability affects external funding and consequently reliable drug supply. A vicious spiral emerges when bypassing weak systems with parallel systems causing further weakening causes of the primary system.

Despite the intention to integrate ARV supply chains with essential drug systems at a later stage, five years into ART such efforts have not matured. This confirms the general axiom that approaches initially designed as disease and program-specific are not easily joined into sector-wide systems [70]. Systems issues rooted in weak governance and disconnected processes are difficult to remedy. Given the nature of reinforcing effects, the dynamics that create adverse effects will accelerate as scale-up, the number of disease-specific interventions, structures and external actors increase. Moreover, new systems become resistant to change as actors develop competing interests, such as remaining employed by new programs. Dynamics thus need to be anticipated and mitigated at early stages. Systems thinking is a way to account for multiple, reinforcing and unpredicted ways in which ART supply chains interact with other health system components. As highlighted by WHO, "a system's failure requires a system's solution - not a temporary remedy" [71]. At the moment, the term "system strengthening" is being largely misused for interventions that continue to have fragmenting effects. Crucially, systems approaches need to tackle the diverse bottlenecks this study has described across building blocks. Important elements include better integration of donors with national structures, long term sustainable funding or improving links between different elements of the health system through regulatory and appropriate feedback systems.

Countries themselves so far have made little use of available funding for health system strengthening [72]. One reason is likely a lack of capacities to develop health system programs with more complex designs as compared to disease-specific interventions. Systems thinking helps countries to assess and appreciate the system effects of interventions and adapt plans accordingly. It helps identify synergistic effects of multiple interventions across the majority of the health system building blocks, with attention to system based monitoring and careful steering of dynamic and interrelated processes. National ownership that allows for continuous follow-up and adaptation as well as the rooting of responses within national institutions therefore constitutes a vital part of any external support.

## Conclusions

This study presents a synthesis of the current way of managing ARV supply in Uganda. It uses the vantage point of a systems thinking lens and a research project which investigates front line provider realities and links them to national developments. It does this through closely examining systems prerequisites in the area of governance, financing, human resource, information and service delivery in general. Its findings identify serious system failures, and dangerous and potentially irreversible dynamics due to the flourishing of disease-specific-intervention and their general focus on short term targets and failure to address current systems bottlenecks. Results are unsatisfactory outcomes not only for HIV but for health in general. The opportunity and need to use ART investments for an essential supply chain management has not been exploited. External aid approaches fail to sustainably strengthen health systems and national responses to disease-specific programs. Shifting to a deeper understanding through systems thinking to shape and continuously follow up interventions that bear potential for system-wide improvements will give better insights to strengthen systems. Key approaches such as long-term funding and targets, evidence-based priority setting and national ownership are largely known. What appears to be missing is the sense of exigency and awareness regarding the risks of not only poor outcomes but system distortions and their hindrance to sustainable progress.

## List of Abbreviations

ART: Antiretroviral therapy; ARV: Antiretroviral drug; CCM: Country Coordinating Mechanism; DHSS: Demographic and health surveillance site; DMO: District medical officers; FBO: Faith-based organization; GFATM: Global Fund to Fight HIV/AIDS, Tuberculosis and Malaria; GHI: Global Health Initiative; HC: Health centre; HIS: Health Information System; IDI: Infectious Diseases Institute; JCRC: Joint Clinical Research Center; JMS: Joint Medical Store; LMIS: Logistics management information system; MAP: Multi-country HIV/AIDS program; MOH: Ministry of Health; MRC: Medical Research Council; MUJHU: Makerere & John Hopkins University Research Collaboration; NGO: Non-governmental organization; NMS: National Medical Store; OI: Opportunistic infections; PEPFAR: United States President's Emergency Plan for AIDS Relief; PIDC: Paediatric Infectious Diseases Clinic; PMTCT: preventing mother-to-child transmission; STI: Sexually transmitted infections; TASO: The AIDS Support Organisation; UAC: Ugandan AIDS Commission; US: United States; VCT: Voluntary counselling and testing

## Competing interests

The authors declare that they have no competing interests.

## Authors' contributions

RW designed the study, performed the analysis and drafted the manuscript. DD contributed to the concept and design of the study, and analysis and drafting of the manuscript. PW participated in the data collection and helped to draft the manuscript. FN designed part of the study and helped to draft the manuscript. FS carried out data collection and participated in the drafting of the manuscript. All authors read and approved the final manuscript.
